# Optimizing Adjuvant Care in Early Breast Cancer: Multidisciplinary Strategies and Innovative Models from Canadian Centers

**DOI:** 10.3390/curroncol32070402

**Published:** 2025-07-14

**Authors:** Angela Chan, Nancy Nixon, Muna Al-Khaifi, Alain Bestavros, Christine Blyth, Winson Y. Cheung, Caroline Hamm, Thomas Joly-Mischlich, Mita Manna, Tom McFarlane, Laura V. Minard, Sarah Naujokaitis, Christine Peragine, Cindy Railton, Scott Edwards

**Affiliations:** 1 Faculty of Medicine, BC Cancer, University of British Columbia, Vancouver, BC V6T 1Z3, Canada; angela.chan@bccancer.bc.ca; 2 Department of Oncology, Cumming School of Medicine, University of Calgary, Calgary, AB T2N 2T8, Canada; nancy.a.nixon@albertahealthservices.ca (N.N.); winson.cheung@oncoutcomes.com (W.Y.C.); 3 Odette Cancer Centre, Sunnybrook Health Science Centre, University of Toronto, Toronto, ON M4N 3M5, Canada; muna.alkhaifi@sunnybrook.ca (M.A.-K.); christine.peragine@sunnybrook.ca (C.P.); 4 Faculté de Médecine, Université de Montréal, Montreal, QC H3T 1J4, Canada; alain.bestavros.med@ssss.gouv.qc.ca; 5 Arthur J.E. Child Comprehensive Cancer Centre, Calgary, AB T2N 5G2, Canada; christine.blyth@albertahealthservices.ca (C.B.); cindy.railton@albertahealthservices.ca (C.R.); 6 Department of Medical Oncology, Windsor Regional Hospital, Windsor, ON N8W 1L9, Canada; caroline.hamm@wrh.on.ca; 7 Université de Sherbrooke Cancer Research Institute (IRCUS), Université de Sherbrooke, Sherbrooke, QC J1E 4K8, Canada; thomas.joly-mischlich.ciussse-chus@ssss.gouv.qc.ca; 8 Department of Medical Oncology, College of Medicine, University of Saskatchewan, Saskatoon, SK S7N 5E5, Canada; mita.manna@saskcancer.ca; 9 School of Pharmacy, University of Waterloo, Kitchener, ON N2G 1C5, Canada; tom.mcfarlane@uwaterloo.ca; 10 Department of Pharmacy, Nova Scotia Health, QEII Health Sciences Centre, Halifax, NS B3H 2Y9, Canada; laurav.minard@nshealth.ca; 11 London Health Sciences Centre, London, ON N6A 5W9, Canada; sarah.naujokaitis@lhsc.on.ca; 12 Faculty of Medicine and School of Pharmacy, Memorial University of Newfoundland, St John’s, NL A1B 3V6, Canada

**Keywords:** CDK4/6 inhibitors, early breast cancer, HR+, HER2–, multidisciplinary care models, team-based care, pharmacist-led care, nurse-led care, NP-led care, GPO-led care, adherence management, toxicity management, oncology care optimization, patient-centered care

## Abstract

The adjuvant treatment landscape for hormone receptor-positive (HR+), human epidermal growth factor receptor 2-negative (HER2–) early breast cancer (EBC) is rapidly evolving, with a diverse range of therapeutic options—including endocrine therapies, bisphosphonates, ovarian function suppression, olaparib, CDK4/6 inhibitors, and emerging agents such as immunotherapy. While these advances have markedly improved patient outcomes, they also introduce challenges related to implementation, monitoring, and resource allocation. Notably, therapies like CDK4/6 inhibitors require particularly close monitoring, creating logistical and capacity challenges for medical oncologists, whose workloads are already stretched due to rising cancer incidence and treatment complexities. These challenges underscore the need for innovative care delivery solutions to ensure patients with EBC continue to receive optimal care. This paper offers a comprehensive guide—a playbook—of multidisciplinary-team-based care models designed to optimize adjuvant treatment delivery in EBC. Drawing on real-world evidence and successful applications across Canadian centers, we explore models led by nurses, nurse practitioners (NPs), general practitioners in oncology (GPO), and pharmacists. Each model leverages the unique expertise of its team to manage treatment toxicities, facilitate adherence, and enhance patient education, thereby promoting effective and sustainable care delivery. Importantly, these models are not intended to compete with one another, but rather to serve as a flexible recipe book from which breast cancer care teams can draw strategies tailored to their local resources and patient needs. By detailing implementation strategies, benefits, and challenges—in many instances supported by quantitative metrics and economic evaluations—this work aims to inspire care teams nationwide to optimize the adjuvant management of patients with HR+, HER2– EBC.

## Simple Summary

Breast cancer is the most common cancer among women in Canada, and more patients are living longer thanks to improved treatments. One common type—hormone receptor-positive, HER2-negative early breast cancer—now has several effective therapies, some of which require frequent monitoring and follow-up. This increases pressure on cancer specialists, especially as cancer diagnoses continue to rise. Our study explores how care teams can meet this demand by adopting innovative, team-based care models. These include approaches led by nurses, nurse practitioners, general practitioners in oncology, and pharmacists. We describe how each model functions, how they support patient safety and treatment adherence, and how they can relieve pressure on oncology services. These models are already in use at some Canadian cancer centers and can be adapted based on local needs and resources. Rather than promoting a single solution, we offer a flexible guide for integrating these roles into routine care. By doing so, cancer teams can continue to deliver high-quality, patient-centered care in a more sustainable way as treatment complexity grows.

## 1. Introduction

More than 90% of breast cancer cases are diagnosed at an early stage (stages I–III). Among these, approximately 70–75% are classified as hormone receptor-positive (HR+), human epidermal growth factor receptor 2-negative (HER2–), making it the most common subtype of breast cancer [1,2]. In Canada, approximately 30,800 new cases of breast cancer and 5500 breast cancer-related deaths were projected in 2024, emphasizing the disease’s profound impact on public health and the critical need for effective treatment strategies [3].

In the neoadjuvant or adjuvant treatment of early breast cancer (EBC), therapeutic strategies are tailored to tumor subtype and recurrence risk. In addition to surgery and radiation, systemic therapies are often recommended, with high-risk individuals typically receiving chemotherapy, sometimes in combination with immunotherapy or targeted therapies. For HR+, HER2– EBC disease, treatment has long relied on maintenance endocrine therapy (ET), along with supportive measures such as ovarian function suppression (OFS) in premenopausal women and bisphosphonates in postmenopausal women. More recently, the addition of CDK4/6 inhibitors to ET in select patients has shown significant clinical benefit. However, these agents require individualized toxicity monitoring, frequent laboratory assessments, and sustained adherence support—demands that can strain already stretched oncology services and highlight the need for scalable, team-based care models.

Adjuvant CDK4/6 inhibitors are of particular interest in this context because of the broad patient population that is now considered for these therapies. The intent-to-treat (ITT) populations of the monarchE and NATALEE trials comprised patients with stage II or III EBC [4,5]. Estimates from real-world databases indicate that between 11% and 25% of patients with HR+, HER2– EBC meet the eligibility criteria for the monarchE trial, while 31 to 43% meet the criteria for the NATALEE trial [6,7,8]. Following the promising outcomes of the monarchE and NATALEE trials, strategies will need to be envisioned to manage the increasing number of patient visits and follow-ups associated with these therapies.

The improved disease-free survival rates in EBC with the integration of adjuvant CDK4/6 inhibitor therapy into clinical practice contribute to a rising workload for medical oncologists who are also facing a rising prevalence of cancer cases globally. Although care teams can draw parallels with other systemic therapies like the combination of trastuzumab and ET for HR+, HER2+ patients in terms of care coordination, adjuvant CDK4/6 inhibitors require more resource-intensive and structured follow-up protocols to manage toxicities and ensure adherence. With CDK4/6 inhibitors, patients are typically followed every 2 weeks for the first 2 months, monthly from months 3–6, then every 3 months until the end of treatment. Close monitoring in the first 6 months is critical, as most treatment-related adverse events occur early during the treatment course. This raises concerns about the sustainability of healthcare systems due to escalating resource demands and workforce shortages, particularly in medical oncology [9]. Combined with the fact that breast cancer leads the projected cancer incidence trends in 2025 [10], innovative strategies are urgently needed to address these challenges and ensure efficient cancer care delivery. Adoption of innovative care models, including nurse-led, nurse practitioner (NP)-led, general practitioner in oncology (GPO)-led, and pharmacist-led models, offers an opportunity to address these challenges by leveraging multidisciplinary approaches to improve patient outcomes, optimize resource allocation, and enhance the sustainability of care delivery systems.

Nurse- and NP-led care models have emerged as effective approaches to enhance patient outcomes and streamline healthcare delivery by leveraging advanced clinical expertise, fostering interdisciplinary collaboration, and ensuring comprehensive, patient-centered care coordination. NP-led care models, with their advanced clinical autonomy, can independently manage stable patients and escalate care when needed. Evidence suggests that NP-led clinics significantly improve patient satisfaction, reduce wait times, and enhance therapeutic relationships, contributing to overall care efficiency [11,12,13]. NP-led survivorship programs in oncology have effectively managed treatment-related adverse events, enhanced quality of life, and facilitated safe transitions to primary care [14]. Similarly, nurse-led interventions, such as telephone triage, post-discharge follow-ups, and telehealth visits, play a critical role in reducing emergency department (ER) visits and hospital readmissions by enabling early detection of symptoms and oncologic emergencies, thereby facilitating timely referrals and interventions [15].

GPO-led care models address the growing demand for oncology services by bridging primary care and oncology, offering sustainable solutions to the increasing cancer incidence and the growing demand on workload with complex targeted therapies [16,17,18]. These models effectively reduce oncologists’ workload by managing systemic treatments, acute and long-term adverse events, and survivorship care. Exemplified by Cancer Care Manitoba, where GPOs provide 28.5% of intravenous chemotherapy annually, GPO-led initiatives enhance accessibility, continuity, and quality of cancer care, especially in underserved regions [18]. Expanding these models could help enhance patient-centered cancer care delivery while preparing healthcare systems for rising oncology demands.

Pharmacist-led care models can leverage pharmacists’ expertise in medication management to monitor adherence, manage toxicities, and provide patient education. Pharmacist-led care models in oncology and beyond have demonstrated significant improvement in symptom scores, patient confidence, and reductions in hospital readmissions by addressing medication-related problems, particularly among patients with polypharmacy, while maintaining high adherence rates [19,20,21,22,23]. A systematic review of 66 studies demonstrated that oncology pharmacists add significant value by enhancing clinical care, improving patient education, advancing informatics, and achieving cost savings, underscoring their essential role as frontline medication experts on the oncology care team [24].

Economic evaluations further support the adoption of these models. Cost analyses have revealed that involvement of full-time pharmacists in a U.S. ambulatory cancer center resulted in an estimated cost avoidance of USD 282,741 per year [24,25]. The Australian, nurse-led Symptom and Urgent Review Clinic (SURC) for patients with cancer achieved a six-month net cost savings of AUD 37,090 and an ROI of AUD 1.73 per dollar invested by reducing ER presentations and inpatient admissions, highlighting its strong financial benefits [26]. These multidisciplinary care frameworks not only optimize medication use and adherence but also reduce hospital admissions and treatment delays, further substantiating their economic benefits in improving healthcare outcomes [16,20,27,28]. These precedents illustrate the feasibility, effectiveness, and economic benefit of multidisciplinary approaches in managing complex care needs. Given the shared complexities of managing HR+, HER2– EBC, particularly with the integration of CDK4/6 inhibitors, these models hold significant potential for improving outcomes.

### Objective

This manuscript aims to provide a detailed exploration of multidisciplinary care models designed for patients with higher-risk HR+, HER2– EBC who require therapies with more intensive monitoring, such as adjuvant CDK4/6 inhibitors. Many of the strategies discussed may also be broadly applicable to patients receiving long-term ET alone. Drawing from evidence-based practices and expert-driven innovations across Canada, the manuscript highlights the roles, implementation strategies, and outcomes of existing pharmacy-, NP-, and GPO-led care models to inspire other EBC care providers to adopt similar innovative approaches. We emphasize that a one-size-fits-all approach to care is insufficient; the optimal model depends on the unique needs of each healthcare center and the individual requirements of each patient, emphasizing the importance of tailoring strategies to local resources, infrastructure, and patient-specific factors.

## 2. Nurse and NP-Led Care Models

### 2.1. Overview

Nurse-led and NP-led care models are vital components of multidisciplinary cancer care, each offering distinct strengths for varying clinical needs. Nurse-led models, supervised by oncologists or NPs, follow strict, evidence-based protocols to handle routine tasks like care coordination, symptom management, patient education, and scheduling follow-ups for stable patients [29,30]. In most jurisdictions, they do not have prescription authority or the capacity for detailed physical examinations necessary for assessing disease progression. In contrast, NP-led models operate with greater autonomy, allowing NPs to conduct advanced assessments, interpret diagnostic tests, and adjust medications, such as CDK4/6 inhibitors, to independently manage complex cases and toxicities [31,32,33].

These clinics have proven effective in diverse areas, including breast and colorectal cancer, by managing symptoms, reducing distress, and achieving patient satisfaction rates exceeding 85% [29,34,35,36]. They also efficiently manage adverse events, reducing ER visits by up to 50% [29,35]. High recruitment and response rates to patient-reported outcomes (PROs) indicate that specialist nurse-led follow-ups are feasible and acceptable for patients with EBC [36]. A survey revealed that oncologists highly valued the skills and expertise of clinical nurse specialists and felt confident referring patients to nurse-led consultations [37]. Nurses have also reported that nurse-led clinics benefit themselves, patients, and healthcare systems [38]. By alleviating oncologist workloads without compromising outcomes, these models offer scalable solutions for various cancer care settings.

NP-led clinics represent a significant advancement in the management of EBC, particularly with the incorporation of adjuvant CDK4/6 inhibitor therapies, meeting the increased demand for intensive monitoring of toxicities and adherence.

### 2.2. What Are Some Examples of Nurse- and NP-Led Care Models?

Nurse- and NP-led care models have been effectively implemented across academic and community settings in Canada. These models provide specialized support for patients undergoing adjuvant therapies, such as CDK4/6 inhibitor treatments, through adaptable approaches tailored to diverse clinical environments and patient needs. The following examples demonstrate their effectiveness and versatility in optimizing oncology care.

**Figure 1:**
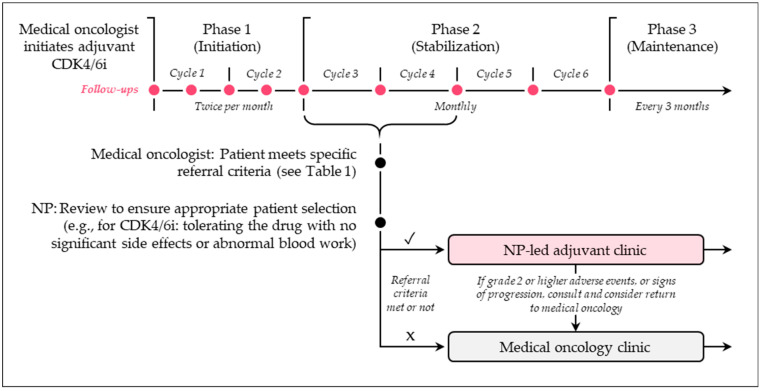
Referral pathway for the adjuvant NP clinic at the Verspeeten Family Cancer Centre (London, ON). Pathway for adjuvant CDK4/6i therapy is depicted. Abbreviations: CDK4/6i = cyclin dependent kinase 4 and 6 inhibitor; NP = nurse practitioner.

At the Verspeeten Family Cancer Centre, an academic hospital in London, Ontario, the implementation of NP-led clinics for adjuvant ET, established through the Participatory, Evidence-based, Patient-focused Process for Advanced Practice Nursing (PEPPA) framework from Cancer Care Ontario, provided a foundation for a new NP-led clinic to manage adjuvant therapies, including CDK4/6 inhibitor therapy [13]. A key component of the business case presented to hospital administrators for funding the new clinic was the estimated number of annual patients with EBC who would qualify for adjuvant CDK4/6 inhibitors, projected to be 250 patients each year. Patient monitoring for adjuvant CDK4/6 inhibitors is structured in three phases: Phase 1 (initiation) involves two follow-ups per month during the first 2 months; Phase 2 (stabilization) includes monthly follow-ups from months 3 to 6; and Phase 3 (maintenance) consists of follow-ups every 3 months after 6 months (Figure 1). A patient is deemed suitable for referral to the NP-led clinic if they meet the four criteria outlined in Table 1. These include receiving one of the specified adjuvant treatments, having a history of invasive breast cancer, an ECOG performance status of 0 to 1, and tolerating treatment well. For those on adjuvant CDK4/6 inhibitor therapy, referrals are permitted after two to three cycles, provided the patient is tolerating the drug without significant adverse events or abnormal bloodwork. The referring medical oncologist submits a physical paper referral to the NP for review to ensure appropriate patient selection. Patients experiencing grade 2 or higher adverse events (e.g., grade 2 diarrhea requiring dose adjustment) during the initiation phase remain in the medical oncology clinic until stabilized. If such adverse events occur in the NP-led clinic, the NP collaborates with the primary medical oncologist to determine whether the patient should continue in the NP-led clinic or return to the medical oncology clinic. This process ensures that only stable, appropriate patients are transitioned to the NP-led clinic for continued care.

Nurse/NP-led care models at the Arthur J.E. Child Comprehensive Cancer Centre in Calgary, Alberta, an academic teaching hospital, have been instrumental in addressing the growing demands of EBC care. One clinic conducts initial medical oncology consultations under very strict criteria for patients with low-risk, node-negative EBC eligible for ET. This innovative approach has been well-received by both patients and medical oncologists, effectively decanting patients from the medical oncology team during periods of lengthy waitlists. Another clinic provides support for patients undergoing adjuvant ET, zoledronic acid, and OFS.

These clinics are staffed by nurses and NPs who receive specialized oncology training, including updates on breast cancer treatment guidelines, pharmacology, and the management of adverse events. Nurse decision-making is protocol-driven, adhering to strict evidence-based guidelines, with deviations requiring consultation with an NP, GPO, or medical oncologist. NPs independently assess patients, adjust doses, and manage complex cases, ensuring timely interventions and seamless communication with oncologists. Transitioning to NP-led clinics requires that patients have no grade 3 adverse events or manageable ones from adjuvant therapy, and that they show adherence to and engagement in their care plan. These clinics employ evidence-based protocols for routine lab monitoring, toxicity management, and psychosocial support. Escalation protocols ensure that severe toxicities (grade 3 or higher) or signs of disease progression are promptly referred to the medical oncologist for further management.

### 2.3. What Are the Benefits and Challenges of Nurse- and NP-Led Care Models?

Nurse-run clinics are cost-efficient and ideal for resource-limited settings, while NP-run clinics enhance care through flexible decision-making and comprehensive service delivery, reducing delays and multiple appointments. A hybrid model, using nurse-led clinics for stable patients and NP-led clinics for complex cases, may offer an optimal balance for many institutions.

Patients and families might initially hesitate to transition from oncologist-led to NP-led care, but they can be reassured of the significant benefits, which include improved medication adherence, reduced wait times, and a high rate of satisfaction. At the Verspeeten Family Cancer Centre, staff have observed increased patient satisfaction and adherence largely due to personalized, one-on-one care that fosters strong therapeutic relationships and addresses medical, psychosocial, and emotional needs. These models alleviate capacity constraints by offloading routine follow-ups from medical oncologists, allowing them to focus on complex cases. The use of standardized, evidence-based protocols ensures consistent, high-quality adverse-event management, leading to fewer complications, reduced hospitalizations, and cost savings. Additionally, these models promote trust between nurse/NPs and medical oncologists and enhance role satisfaction and retention among nurse/NP staff.

Challenges include securing financial and institutional support for infrastructure, such as dedicated space, as well as staffing and training. Maintaining appropriate nurse-to-patient ratios to prevent staff overload is critical. Transitioning patients from oncologist-led to nurse-/NP-led care requires clear communication for patient and family acceptance and effective coordination among healthcare providers for timely escalations. Additional challenges include slow referral initiation and difficulty estimating patient volumes due to varying treatment-based follow-up needs. For example, patients on ET and zoledronic acid may require only one to two follow-ups annually, while those on abemaciclib might need up to nine visits per year. Differing levels of comfort and preferences among oncologists, along with ensuring accurate patient bookings, further complicate implementation.

At the Verspeeten Family Cancer Centre, NP-led clinics operate twice per week, with each NP seeing about 10 patients per day. The clinic currently serves 130 patients, with plans to expand clinic days. Operating at approximately 70% capacity, the model will be formally evaluated through measures like disease-free survival and toxicity management rates.

### 2.4. What Are Some Recommendations for Practice?

Nurse and NP-led care models at the Verspeeten Family Cancer Centre and Arthur J.E. Child Comprehensive Cancer Centre emphasize clear roles in patient assessment, adverse-event monitoring, and education. Continuous education and oncology training for nurses and NPs are essential, supported by evidence-based protocols. Collaboration with oncologists, pharmacists, dietitians, social workers, and clerical staff enhances holistic care and service integration.

Regular evaluation with standardized metrics, such as adherence rates and patient satisfaction surveys, supports continuous improvement. Monthly working groups at the Verspeeten Family Cancer Centre optimize workflows and address challenges. Infrastructure improvements, like adequate staffing and electronic medical record (EMR) integration, are critical for sustainability. Oncologists have noted benefits such as easier referral processes and shorter wait times for patients.

Expanding NP-led clinics to community centers and using digital tools like telehealth can improve accessibility and efficiency. These models reduce healthcare costs by allocating routine care to advanced practice providers, reserving specialists for complex cases. This scalable strategy aims to meet growing EBC care demands by reducing oncologist workloads and improving patient outcomes. Securing funding through grants and partnerships supports training and expansion, with pilot programs offering scalable implementation approaches. Many of these strategies align with those outlined for GPO-led care models, as detailed in Table 2.

## 3. GPO-Led Care Models

### 3.1. Overview

GPOs, defined as family physicians with specialized oncology practices, are essential to help meet the growing need for comprehensive cancer care. GPO-led care models have demonstrated the ability to seamlessly integrate primary-care expertise with oncology-specific training across various oncology settings [18].

Although there is limited literature on GPO-led models compared to the roles of NPs and pharmacists, several studies have investigated the transfer of follow-up care for patients with breast cancer from oncologists to family physicians [39,40,41,42,43]. A randomized controlled trial found that breast cancer survivors followed by family physicians had recurrence rates, serious clinical events, and health-related quality of life (HRQL) outcomes similar to those followed in cancer centers [39]. Another study revealed that 43% of EBC survivors were suitable for transition to primary care, while 57% required ongoing oncologist oversight due to clinical trial participation, ongoing ET, new symptoms, or personal preference [40].

Patient satisfaction with family-physician-led follow-up has been well documented [41,42,43]. In a survey of low-income, medically underserved patients with breast cancer, 73.4% reported high satisfaction with their follow-up care, citing the ability to ask questions and clear communication as key factors [41]. Another survey showed that while 74% of breast cancer survivors felt reassured by hospital-based follow-up, 67% were confident in family physician-led survivorship care [43].

These findings highlight the potential of GPO-led models to deliver high-quality, seamless, and sustainable cancer care. By providing structured follow-up care, GPOs can ensure adherence to adjuvant therapies, monitor for recurrence, manage treatment-related adverse events, and maintain effective communication, ultimately improving long-term outcomes for patients with EBC.

### 3.2. What Are Some Examples of GPO-Led Care Models?

GPO-led care models have been successfully implemented across Canada, demonstrating their adaptability and impact on patient outcomes.

The Breast Cancer Follow-up and Survivorship Program at the Louise Temerty Breast Cancer Centre, Sunnybrook Hospital in Toronto, Ontario, offers follow-up care for patients with breast cancer who have completed active treatment, such as radiation and chemotherapy, and have no evidence of disease (12–18 months post-surgery), as well as those receiving ongoing adjuvant therapy [44]. This GPO-led program addresses the unmet need for survivorship care by managing long-term treatment effects and facilitating the transition to primary care when appropriate. Operating three times weekly, both in-person and virtually, the clinic serves approximately 40–50 patients per session.

**Figure 2:**
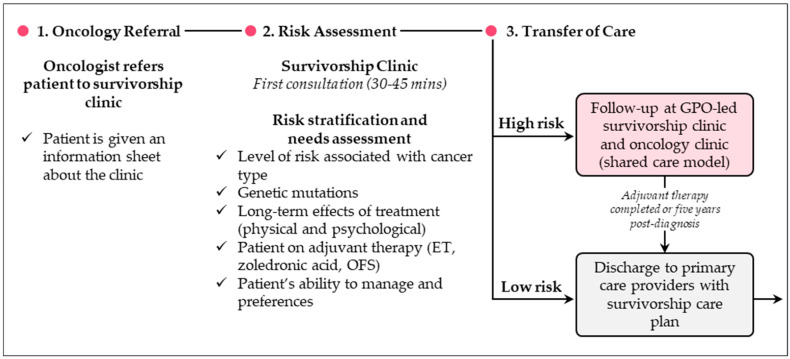
Overview of survivorship care process at the Louise Temerty Breast Cancer Centre Survivorship Clinic (Toronto, ON). Abbreviations: ET = endocrine therapy; GPO = general practitioner in oncology; OFS = ovarian function suppression.

The clinic offers a standardized, comprehensive, evidence-based, and stratified approach to survivorship care, facilitating the transition of breast cancer survivors to primary care. During the initial consultation, patients undergo cancer recurrence surveillance, receive guidance on managing cancer-related effects, and complete a comprehensive risk assessment (Figure 2). They are also counseled on lifestyle modifications. This assessment evaluates factors such as cancer type, genetic mutations, treatment adverse events, and preferences for ongoing care. Patients are categorized as “low risk” or “high risk”. “Low risk” patients are discharged to their primary-care provider with a tailored Survivorship Care Plan (SCP), which is shared with their family physician to support long-term follow-up care. “High risk” patients—those with genetic risks, ongoing adverse events, high-risk imaging needs, psychosocial challenges, or lack of access to primary care—require continued follow-up at the clinic. For patients on adjuvant ET, discharge to primary care is considered after completing treatment or five years post-diagnosis.

Additionally, the clinic provides access to a wide range of multidisciplinary services within the Louise Temerty Breast Cancer Centre, including physiotherapy, psychotherapy, spiritual care, dietitians, lymphedema management, palliative care, and art therapy for patient and family support. In partnership with Wellspring, an organization dedicated to supporting cancer survivors and their families, the clinic has expanded its educational offerings, including a recent series of webinars that enhanced participant knowledge of cancer survivorship [45].

In Calgary, Alberta, the Arthur J.E. Child Comprehensive Cancer Center includes a GPO-led clinic that manages patients with breast cancer who are eligible for adjuvant ET plus CDK4/6 inhibitor therapy or adjuvant olaparib. A medical oncologist initiates the treatment, and once it is well-tolerated, they transition to GPO-led care. Because GPOs are active in medical oncology clinics, many patients are already familiar with them, making the transition smoother. The clinic operates independently of medical oncologist supervision, addressing any physical concerns during appointments and providing reassurance or ordering further investigations as needed. Patient volume is expected to increase due to expanded eligibility from the NATALEE trial with ribociclib plus ET, which included a broader population of patients with high-risk stage II or III EBC compared with the monarchE trial with abemaciclib [4,5].

Operating within Alberta Health Services (AHS), the clinic benefits from resources such as clinic space, nursing support, and pharmacist support. The clinic operates for a half-day during the first, third, and fifth weeks of the month, serving 8–15 patients per session. Patients begin therapy with their oncologist and have monthly follow-ups for the first 6 months. After that, follow-ups may change to every 3 months for the rest of their planned 2-year treatment with abemaciclib or 1 year with olaparib. About 90% of visits are in-person, with virtual visits accounting for less than 10%. Patients can alternate visits with their medical oncologist based on preference.

GPOs at the clinic manage therapy adherence, toxicity, and adverse events, with authority to adjust dosages, schedules, and supportive medications as necessary. Patients undergo routine cancer surveillance and can be referred to specialists, psychosocial support, or dietitians. All progress notes are recorded in the EMR and shared with the referring oncologist and family physician. After completing therapy, patients may return to their oncologist or transition to community care, depending on recurrence risk, long-term adverse events, and ongoing therapy needs such as bisphosphonates or OFS.

### 3.3. What Are the Benefits and Challenges of GPO-Led Care Models?

Although formal PROs have not been collected, providers at the Arthur J.E. Child Comprehensive Cancer Center report high subjective patient satisfaction. Their GPO-led clinic aims to evaluate success through metrics such as patient safety, adherence, quality of life, and healthcare resource utilization. A pilot project is underway to systematically assess these factors and provide actionable insights.

With the GPO-led care model, patients benefit from personalized, consistent care from GPOs familiar with their medical history and treatment journey. These models have the potential to reduce the workload of medical oncologists by managing routine follow-ups for stable patients, allowing oncologists to focus on complex cases. Additionally, GPO-led clinics can improve access to care, especially in rural and underserved areas, through telehealth integration and community-based approaches. However, a significant challenge remains: the availability of trained GPOs.

GPO-led clinics often serve as academic hubs, providing education and training to family medicine residents and other healthcare professionals. Initiatives such as didactic sessions, online modules, reflective sessions, and patient-led sessions at the Louise Temerty Breast Cancer Centre educate family medicine residents about cancer survivorship. Pre- and post-program evaluations have shown that these sessions improve residents’ attitudes and knowledge of cancer survivorship. Plans to expand educational programs to other academic centers and national platforms aim to broaden their reach and impact.

### 3.4. What Are Some Recommendations for Practice?

To effectively implement a GPO-led care model, several strategic steps should be considered, as outlined in Table 2. Key recommendations include establishing clear referral pathways and risk criteria; enhancing patient education; standardizing clinic structures and follow-up schedules; optimizing workflow and resource allocation; and implementing robust quality assessment measures. These steps aim to ensure smooth patient care transitions, improve patient outcomes, and enhance the quality and accessibility of survivorship care for patients with breast cancer.

## 4. Pharmacist-Led Care Models

### 4.1. Overview

Pharmacist-led care models in oncology are crucial for managing the increasing complexity of treating patients with EBC who are receiving oral anticancer medications (OAMs), including CDK4/6 inhibitor therapies. These models utilize pharmacists’ expertise to enhance medication adherence, optimize treatment outcomes, and improve patient satisfaction, while reducing the workload of medical oncologists [46].

A real-world study in Canada demonstrated that a structured, patient-centered pharmacy model, which integrates American Society of Clinical Oncology (ASCO) and National Community Oncology Dispensing Association (NCODA) standards, achieved high adherence (89.6%) and treatment continuity in patients with advanced breast cancer treated with CDK4/6 inhibitors [47]. Similarly, a pharmacist-led oral chemotherapy program increased patient comprehension from 43% to 95%, improved adherence from 85% to 95%, and achieved an 83% major molecular response rate in chronic myeloid leukemia, surpassing previous benchmarks [48]. A pharmacist-led monitoring program in a genitourinary oncology clinic improved adherence from 61% to 89% [49].

A combined nurse- and pharmacist-led clinic for metastatic colorectal cancer resulted in 79% of patients completing three or more cycles of capecitabine. Retrospective evaluations indicated an 85% patient satisfaction rate [34]. Additionally, a pharmacist-led remote monitoring service for adjuvant abemaciclib effectively managed dose reductions and interruptions, achieving high patient satisfaction [50]. Of the participants, 89.5% recommended electronic PROs, and 98% found bloodwork closer to home (BCTH) convenient. Furthermore, a pharmacist-led monitoring program for patients with metastatic clear cell renal-cell carcinoma receiving sunitinib improved toxicity management through proactive dose adjustments and follow-ups, demonstrating its feasibility for broader oncology applications [51]. Among 56 patients, 39 dose reductions occurred, primarily during the first cycle.

A Canadian survey of 44 oncology pharmacists revealed that 45% of institutions had formal pharmacist-led monitoring programs focusing on chemotherapy order verification, laboratory monitoring, and patient counseling [52]. While 59% of respondents tracked pharmacy performance metrics, none were universal. Nearly all (98%) supported the development of national clinical pharmacy key performance indicators (cpKPIs) to standardize and enhance oncology pharmacy practices.

These findings underscore the expanding role of pharmacist-led oncology care models and their potential for broader implementation across various cancer types and treatment settings.

### 4.2. What Are Some Examples of Pharmacist-Led Care Models?

Pharmacist-led care models have been successfully implemented across Canada, demonstrating their diversity and adaptability in oncology care.

The Sunnybrook Odette Cancer Centre’s OAM program in Toronto, Ontario, is a pharmacist-led initiative aimed at optimizing patient management for those receiving OAMs [53]. A key feature of the program is its proactive follow-up system, where pharmacists and pharmacy students conduct scheduled phone assessments during the first one to two cycles of therapy. This process ensures adherence and addresses any toxicities using detailed in-house algorithms [54]. The program also involves reviewing patient charts, managing drug interactions, and resolving reimbursement issues to maintain uninterrupted treatment. Annually, the program manages over 1000 patient encounters and has achieved a 25% reduction in unplanned healthcare visits, including ER and urgent care visits. Medication adherence has improved by about 15%, and patient satisfaction surveys consistently emphasize the program’s effectiveness in providing timely support [55]. Patients feel well-supported, as they can easily communicate with a member of the oncology team between visits, with concerns addressed within 12 to 24 h if necessary. Pharmacists independently handle over 80% of patient concerns. Supported by revenue from the Odette retail pharmacy, the program is financially sustainable. Its integration with the EMR system enhances cross-specialty care. These elements make the OAM program a leading example of an interprofessional care model.

**Figure 3:**
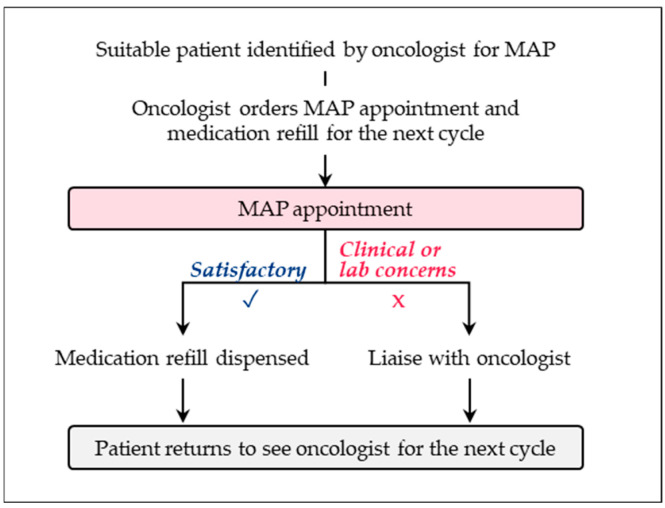
Process overview of Medication Assessment by Pharmacist (MAP) program (Surrey, BC).

The Medication Assessment by Pharmacists (MAP) program at BC Cancer Surrey in Surrey, British Columbia, launched in 2019, integrates oncology pharmacists into a shared care model for patients with metastatic breast cancer receiving a CDK4/6 inhibitor. When treatment is initiated, the medical oncologist orders the medication for one cycle and includes a refill using the standardized provincial order form. The oncologist schedules a medication assessment with the pharmacist for the patient’s next clinic visit, followed by a subsequent appointment with the oncologist (see Figure 3) [56]. During each MAP visit, the pharmacist performs medication reviews, monitors adherence, manages adverse events, and conducts toxicity assessments using standardized tools and provincial protocols. Since its inception, the program has expanded to include 13 OAMs across six malignancies. This expansion now includes patients with EBC treated with adjuvant CDK4/6 inhibitors. According to Chan et al. (manuscript in preparation) [57], it has saved more than 146 h of clinic time through 439 appointments. Patients in the MAP program exhibited higher adherence to provincial and regional protocols for scheduling clinic visits (99%) compared to non-participants (96%). All participating pharmacists (100%) regularly assessed adherence, whereas only 33% of oncologists did [56]. This frequent assessment increases patient interaction opportunities. In 2023, MAP received a Leading Practice Designation from Accreditation Canada, highlighting its success in optimizing pharmacist contributions, reducing oncologist workloads, and maintaining high patient satisfaction. Plans for provincial expansion and the inclusion of additional OAMs underscore its scalability and systemic value.

A pharmacist-led care program at the Dr. H. Bliss Murphy Cancer Centre in St. John’s, Newfoundland leverages pharmacists’ expertise to support medication adherence, manage reimbursement challenges, monitor adverse events, and provide patient education. An assessment of program outcomes revealed that an average of 3.7 drug-related problems (DRPs) were identified and resolved per patient [58]. Surveys across Atlantic Canada show that 58% of oncology practice sites have implemented formal pharmacist-led monitoring programs [59]. Among sites without such programs, staffing shortages and insufficiently trained clinical personnel were the main barriers. Additionally, all respondents (100%) supported establishing formal monitoring programs in hospitals with high volumes of anticancer drug prescriptions. These initiatives have also achieved a 93.6% rate of patient counseling at therapy initiation, enhancing patients’ understanding and adherence to complex regimens.

At the QEII Health Sciences Centre in Halifax, Nova Scotia, a pharmacist-led clinic for patients with (neo)adjuvant breast cancer receiving intravenous therapy was established through multidisciplinary collaboration and proactive stakeholder engagement [60]. An informal assessment revealed significant patient knowledge gaps, securing stakeholder support for pharmacy involvement in education. Support from pharmacy leadership and nursing teams facilitated workflow integration. A small team of pharmacists developed documentation tools, internal education sessions, and a spreadsheet to track clinic impact. Pharmacists in this clinic manage toxicities, provide education, and alleviate physician and nurse workloads through additional visits and follow-ups, especially during initial treatment cycles or regimen changes.

Structured care transitions were also implemented in a pharmacist-led gynecology oncology poly (ADP-ribose) polymerase (PARP) inhibitor clinic at the QEII Health Sciences Centre, with pharmacists managing patients between oncologist visits [61]. Dedicated consultation spaces and after-hours planning with gynecology–oncology teams ensured seamless implementation. Pharmacists working in this clinic conduct toxicity assessments, review bloodwork, manage toxicities, prescribe medications, and provide patient education. After two years of clinic implementation and 601 patient encounters, pharmacists identified 278 DRPs, made 131 clinical interventions, 148 interventions related to the coordination of care, and offloaded approximately two-thirds of physician/nurse patient encounters [61]. The team was recognized nationally for their innovative work, earning the HOPE (Honouring Oncology Pharmacy Excellence) Award [62].

Similarly, a pharmacist-led ambulatory program, the Prostate Cancer Shared Care Pharmacy Clinic, was implemented at the QEII Health Sciences Centre using four iterations of the Plan-Do-Study-Act (PDSA) quality improvement framework [63]. All patients with prostate cancer receiving OAMs were eligible for referral to the final version of the clinic. Integrating the referral process into the existing workflow streamlined patient referrals, while defined practice models and metrics tracking supported program evaluation, provision of clinical services, and capacity assessment. The co-location of pharmacists with the oncology team enhanced relationship building and facilitated efficient collaborative care. Patients in the program alternated visits with the pharmacist and the oncologist/nurse team, which helped redistribute workloads and increase clinic capacity to address growing patient waitlists and ongoing care needs. Between July 2019 and January 2023, the pharmacist saw 136 patients, resulting in 464 total encounters and the identification of 257 DRPs (84% resolved by the pharmacist). Clinical interventions included patient education, supportive care, toxicity management, adherence assessments and support, and deprescribing.

### 4.3. What Are the Benefits and Challenges of Pharmacist-Led Models?

Integrating pharmacists into shared care models reduces ambulatory visits for oncologists while ensuring compliance with provincial monitoring guidelines for patients on OAMs. These models improve adherence, enhance patient education, and decrease healthcare utilization [56]. Patients report high satisfaction with the personalized attention and support provided by pharmacists [55]. A significant benefit in the context of adjuvant CDK4/6 inhibitor therapy is the pharmacist’s ability to conduct comprehensive medication reviews, mitigating potential drug–drug interactions. Additionally, these models lighten the workload of oncologists by allowing them to concentrate on complex cases. Pharmacists’ expertise in reimbursement processes minimizes treatment delays and ensures timely access to therapies [64].

Pharmacist-led programs have shown reduced unplanned healthcare visits, including ER admissions. Pharmacists in initiatives like the OAM program at Sunnybrook Odette Cancer Centre and the MAP program at BC Cancer Surrey resolve over 75% of patient concerns, demonstrating their efficiency in managing toxicity and adherence. As mentioned above, pharmacists at the Dr. H. Bliss Murphy Cancer Centre resolved an average of 3.7 DRPs per patient, with higher intervention rates in the adjuvant setting (4.1 DRPs per patient) compared with the metastatic setting (2.6 DRPs per patient) [58].

However, challenges remain. Key obstacles include variability in referral processes, infrastructure limitations, and maintaining suitable pharmacist-to-patient ratios. Staffing shortages, worsened by rising demand for oncology services, are significant hurdles. Additionally, pharmacists do not conduct physical examinations and cannot order diagnostic imaging, though they can coordinate with physicians and recommend tests. Regulatory constraints often limit pharmacists’ ability to modify treatment plans independently, while digital integration inefficiencies hinder documentation and care coordination.

For instance, the MAP program faces challenges such as limited clinic space, scheduling difficulties within new EMR systems, and a lack of provincial integration into order sets. Similarly, Sunnybrook’s OAM program experiences fragmented care due to unclear communication and duplicated efforts, as well as limitations in visual assessments because of reliance on telephone interactions. At the QEII Health Sciences Centre, pharmacist-led models struggle with inefficiencies related to paper-based documentation systems, outdated order sets, and coordination challenges that delay access to patient charts and timely drug procurement. Additionally, limited opportunities for sharing clinic spaces with other members of the multidisciplinary team further impede optimization.

Addressing these challenges requires targeted investments in training, staffing, physical space, and digital infrastructure. Enhanced toxicity assessment programs can mitigate adverse events and ensure safer treatments. Pilot initiatives using quality improvement frameworks emphasize the need for iterative program development to refine service delivery and optimize resources.

### 4.4. What Are Some Recommendations for Practice?

To optimize pharmacist-led care models, several strategic steps should be considered, as outlined in Table 3. Key recommendations include defining clear roles for pharmacists, implementing proactive care interventions, fostering collaboration among healthcare professionals, improving infrastructure, conducting regular evaluations, and expanding pharmacists’ prescribing authority. These steps aim to enhance patient care quality, streamline workflows, and improve overall patient outcomes in oncology care.

## 5. Digital Health Tools

### 5.1. Overview

**Figure 4:**
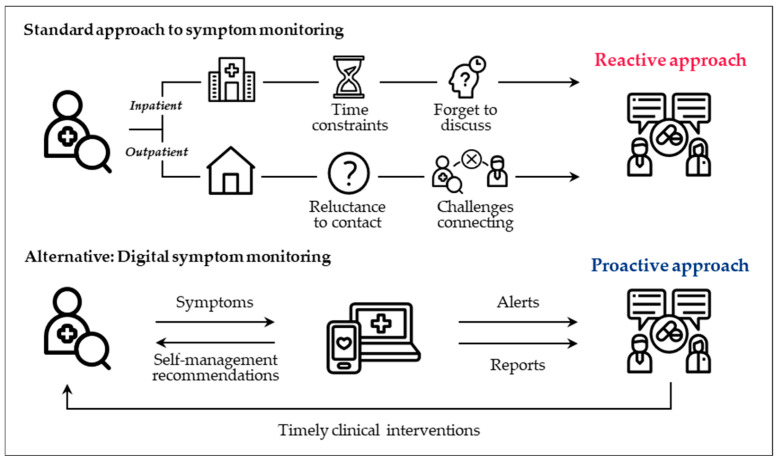
Symptom monitoring: standard versus digital approaches.

Digital symptom monitoring via electronic PROs can significantly enhance cancer care by improving symptom management, patient engagement, and clinical decision-making [65,66,67]. Symptoms often go undetected by providers who rely on standard monitoring approaches, creating a gap that digital monitoring can effectively fill (Figure 4) [68,69,70]. Studies have shown that digital symptom-monitoring tools lead to better outcomes for patients on systemic anticancer therapies, including improved symptom control and quality of life, fewer ER visits, longer treatment duration, and increased survival rates [71,72,73,74,75]. Adoption (85%) and adherence rates (76%) to digital tools are notably high [65].

These tools enhance communication between patients and healthcare providers and offer easy access to educational resources, promoting effective teamwork among care team members [67]. Best practices recommend using a concise questionnaire targeting 10 to 15 actionable symptoms, administered one to three times per week [73]. Patients report symptom intensity, duration, and impact, while some tools can provide educational resources such as self-management guides and symptom diaries, empowering patients and ensuring continuous clinical oversight. Automated alerts trigger timely interventions from healthcare professionals when needed.

The questionnaire should be accessible via web, smartphone, or automated telephone interfaces and can be integrated with EMR systems or operate independently.

Upon initiating anticancer therapy, a symptom-monitoring team supports patients with virtual education and platform guidance. Patients, or their caregivers, should be comfortable with the technology, and those consenting receive additional training. Digital monitoring complements medical follow-ups and may reduce appointment frequency as the system becomes established. All clinical interventions are documented and accessible to oncology teams, enhancing coordinated care.

### 5.2. What Are Some Implementation Examples of Digital Health Tools?

The Suivi Actif des Résultats rapportés par le patient en Oncologie—Médicaments Antinéoplasiques administrés par Voie Orale (SARO-MAVO) project, implemented at CIUSSS de l’Estrie-CHUS in Quebec, addresses a critical gap in oral oncology care by introducing a virtual home-based monitoring platform for patients on OAMs [76]. This initiative enhances symptom monitoring, early intervention, and care coordination through real-time tracking of treatment-related toxicities. Previously, long-term symptom monitoring was managed by community pharmacists and primary-care physicians who often lacked specialized expertise, leading to delayed care and unnecessary ER visits. The SARO-MAVO platform resolves these issues with automated symptom triage, alerts, and tailored medical guidance, allowing timely interventions by oncology pharmacists.

A 1-year pilot study with 227 patients demonstrated high engagement and clinical efficiency (unpublished). Sixty percent of eligible patients participated in virtual follow-ups, and 50% actively used the platform. Among users, 66% triggered symptom alerts, resulting in 760 clinical interventions, primarily through direct patient interactions, with only a small percentage requiring specialist referrals or emergency care. This led to significant improvements in quality and safety, including reduced ER visits and enhanced patient autonomy.

Despite challenges such as cultural resistance, resource limitations, reimbursement issues, and technological barriers, the project succeeded due to strong organizational commitment, local leadership, and clinical champions. The model demonstrated scalability, with collaborations across Quebec and the rest of Canada, and received provincial support for broader implementation. It sets a new standard in virtual oncology care and has the potential to extend to other treatments requiring continuous monitoring. Upcoming studies will evaluate the feasibility and impact of telehomecare monitoring in cancer care, focusing on implementation feasibility, PROs, and equitable access [77].

### 5.3. What Are the Benefits and Challenges of Digital Health Tools?

Digital health tools enhance cancer care by improving communication, education, and monitoring, leading to better patient outcomes and quality of life, while empowering patients and healthcare professionals. These tools enable patients to manage their symptoms proactively, offering tailored recommendations and notifying clinical teams when interventions are needed. This patient-centered approach reduces unnecessary disruptions and improves survival outcomes by focusing on patients who need support [71,72,73,74,75]. It also alleviates the workload of oncologists by delegating symptom monitoring to clinicians using the platform.

However, challenges remain, such as ensuring access to dedicated healthcare providers to monitor alerts amid labor shortages and perceived additional expenses. Organizational commitment is necessary to reallocate resources and implement the model without adding extra staff. Integration with clinical records is another hurdle, requiring manual data transcription. Additionally, some patients, such as the elderly, those with limited digital literacy, or individuals facing socioeconomic barriers, may have difficulty accessing these services. Addressing these issues with planning and investment is crucial.

To ensure sustainability, it is vital to evaluate the model’s impact on patients, professionals, and the healthcare system. Understanding variations across urban and rural settings and staffing levels is important. An evaluability study has been conducted to assess these factors, focusing on implementation context, the effectiveness of electronic patient-PROs and experiences, and model feasibility [77]. These insights will guide evidence-based recommendations for province-wide implementation, ensuring adaptability and scalability across diverse healthcare settings.

### 5.4. What Are Some Recommendations for Practice?

To effectively implement active symptom monitoring utilizing PROs, several key strategies should be considered (Table 4). These include fostering a proactive management culture, designating dedicated resources and clinical champions, ensuring access to telecare technology, securing organizational support, allowing local model adaptation, and conducting ongoing evaluation. It is crucial that these tools enable patients to accurately report the severity of their symptoms, allowing the healthcare team to promptly and appropriately respond according to the grade of the adverse event. These steps aim to enhance patient care quality and optimize oncology healthcare delivery.

## 6. Discussion

The landscape of EBC management is evolving, particularly with the introduction of therapies such as adjuvant CDK4/6 inhibitors. This evolution prompts a critical need for innovative care models to effectively manage the rising burden on medical oncologists. Pharmacist-, nurse/NP-, and GPO-led care models present promising approaches that can optimize resource allocation, enhance patient care, and contribute to the sustainability of oncology services. A summary of the innovative care models discussed is provided in Table 5.

### 6.1. How Should Patients Be Transitioned to an Innovative Care Model?

Transitioning patients to innovative care models should be a carefully considered process, tailored to individual patient profiles and institutional resources. Factors such as performance status, comorbidities, risk of drug interactions, health literacy, decision-making ability, and personal preferences are crucial in determining the suitability of patients for these models. For instance, patients receiving adjuvant CDK4/6 inhibitor therapy may be transitioned soon after treatment initiation or once they are considered stable on their treatment regimen. The definition of “stability” can vary among providers and institutions, but generally, patients are deemed stable after two to three cycles of adjuvant CDK4/6 inhibitor therapy if they tolerate the medication without significant adverse events, abnormal bloodwork (i.e., grade 2 or higher), or dose modifications. The timing of transitioning to an innovative care model will depend on provider and institutional preferences. For example, a well-supported model with providers who have a broad scope of practice may manage early-onset toxicities effectively. Regardless of preferences, clear referral protocols are recommended to guide providers. Candidate patients may greatly benefit from non-traditional care models, while those with significant medical complexities might remain under the direct care of medical oncologists.

While this manuscript emphasizes the application of innovative care models for higher-risk patients receiving therapies such as adjuvant CDK4/6 inhibitors, many of the same principles—including patient education, standardized referral protocols, and interdisciplinary follow-up—are equally applicable to patients with lower-risk HR+/HER2– disease who are receiving ET alone. Given the long duration of adjuvant ET and its associated adherence and toxicity challenges, extending these models to this broader population may enhance continuity of care and improve long-term outcomes.

Both published evidence and our own experience suggest that robust referral protocols, coupled with clear communication and patient education, are essential to facilitate these transitions to innovative models of care [78,79,80,81]. Poor coordination of care during transitions can lead to medication errors, missed adverse events, increased ER admissions, and decreased trust of the patient [78]. To address these challenges, several practical solutions can be implemented. These include clear referral pathways, defined scopes of practice for each care provider, and explicit guidance on when to consult the primary oncologist. Tools that support communication—such as shared EMRs, interdisciplinary case reviews, and standardized documentation—can further enhance coordination and reduce ambiguity in roles and responsibilities. When combined with tailored patient education at the point of transition, these strategies can help overcome many of the limitations associated with decentralized care, ensuring smoother implementation across diverse clinical settings. Studies have shown that robust transitional care can significantly reduce readmissions and ER visits, and cut healthcare costs [82,83,84].

### 6.2. What Should Be the Role of the Medical Oncologist?

Integrating innovative care models into oncology practice does not diminish the critical role of medical oncologists. Their expertise remains invaluable in initiating treatment, managing complex cases, and addressing severe toxicities. A survey found that transitioning stable patients with breast cancer to community care enhanced the efficiency and sustainability of medical oncologists’ practices [85]. In our experience, non-oncologist care providers such as nurses, NPs, GPOs, and pharmacists can effectively manage routine follow-ups, toxicity monitoring, protocol-mandated dose reductions, and medication switches within the same class for tolerability (e.g., letrozole to anastrozole). This delegation of tasks allows oncologists to focus on complex clinical decisions and high-priority cases. Furthermore, some centers benefit from alternating follow-up visits between the oncology clinic and the innovative care model, while others escalate patients back to oncologists only for complications. Oncologists should also be consulted if a patient wishes to discontinue treatment or if there is suspicion of disease recurrence. Reliable communication channels, such as integrated EMRs, can facilitate seamless coordination with care teams.

Strong leadership from oncologists, particularly in collaborating to develop clear protocols that define when their input is necessary, ensures that these innovative models operate effectively. This approach prevents the overburdening of oncologists with routine queries and allows them to concentrate on critical decision-making processes.

### 6.3. What Are Some Recommendations for Monitoring and Managing Adverse Events?

Effective management of adverse events within innovative care models involves leveraging the expertise of nurses, NPs, GPOs, and pharmacists for early intervention. This is particularly important for addressing toxicities such as neutropenia and fatigue, common to both abemaciclib and ribociclib, and diarrhea, which is specific to abemaciclib. Oncologists can then reserve their involvement for the management of severe or uncommon toxicities. Digital monitoring tools, such as electronic PROs, can significantly enhance the early detection and management of adverse events, ultimately reducing unplanned ER visits and hospital admissions [77,86]. Additionally, standardizing medication review and patient education during transitional care has been associated with improved adherence and safety, underscoring the importance of these processes in managing adverse events effectively.

### 6.4. How Do We Ensure Quality of Care?

Ensuring the quality of care in innovative models requires a multi-faceted approach. Monitoring key indicators such as patient satisfaction, medication adherence rates, adherence to treatment protocols/guidelines and frequency of ER visits is crucial. Clinician satisfaction was also highlighted as a key metric, ensuring that care team members feel supported in their roles. Interdisciplinary collaboration and clear escalation pathways are essential in maintaining high standards of care. Regular quality assessments and prospective studies can provide valuable insights into the long-term clinical and economic impacts of these models, ensuring they remain aligned with evolving best practices. A focus on patient education is critical to supporting transitions, helping individuals understand their treatment plans, potential adverse events, and when to escalate concerns. Structured communication training for patients and caregivers has also been shown to improve self-management and reduce hospital admissions [87].

### 6.5. What Are Some Future Directions for Innovative Models of Care?

Looking to the future, expanding interdisciplinary care models and integrating digital health solutions could significantly enhance patient engagement and monitoring. While randomized trials comparing care models are limited, some validation efforts are underway. For example, the MAP program at BC Cancer Surrey is undergoing further study with planned patient surveys, and a recent multi-center cross-cohort study in Atlantic Canada found that patients receiving proactive pharmacist-led monitoring stayed on treatment longer and had fewer severe toxicities and ER visits than those in reactive models [88]. These findings support the potential of structured, pharmacist-led approaches.

Telehealth services and remote adverse-event monitoring/reporting systems hold promise for supporting adherence and toxicity management by providing real-time data to care teams. Additionally, innovations such as smart medication adherence devices, which can track pill usage and send reminders to patients and caregivers, offer exciting opportunities to improve adherence [89]. Programs that incorporate digital tools alongside innovative care models and structured oncology follow-ups may further strengthen patient-centered care and improve long-term outcomes. Furthermore, exploring AI-driven predictive models could allow for earlier interventions by identifying patients at risk for nonadherence or severe toxicities. There is also potential in expanding the roles of physician assistants (PAs) in oncology care, as demonstrated by successful implementations at institutions such as Dana-Farber Cancer Institute [90]. The integration of PAs into cancer care teams has shown improvements in care efficiency, patient access, and satisfaction, offering another avenue to optimize resource utilization while maintaining high-quality care [91,92]. Some jurisdictions are also considering a role for community pharmacists in following patients who are on adjuvant therapies.

## 7. Conclusions

In summary, the integration of pharmacist-, nurse/NP-, GPO-, or PA-led care models in EBC management represents a promising approach to optimizing treatment adherence, minimizing toxicities, and enhancing multidisciplinary collaboration. Expanding these models, with a focus on structured referral processes, clinician support, and digital integration, may further strengthen patient-centered care and improve long-term outcomes for survivors. Each institution can tailor these innovative care models according to their local practices, resource allocations, and the specific needs of their patient populations, ensuring that the implemented strategies are both effective and sustainable.

## Figures and Tables

**Table 1:** Referral criteria for the adjuvant NP clinic at the Verspeeten Family Cancer Centre (London, ON).

Referral Criteria	Yes
1. History of invasive breast cancer	✓
2. ECOG PS 0-1 and tolerating treatment	✓
3. Not on a clinical trial that requires follow-up by Medical Oncology	✓
4. Must be receiving one of the following treatments:	✓
Adjuvant trastuzumab or T-DM1	
Adjuvant capecitabine	
Adjuvant olaparib	
Adjuvant weekly paclitaxel (+/− trastuzumab) or docetaxel and cyclophosphamide after completion of 1 cycle and tolerating well with no significant adverse events	
Adjuvant CDK4/6 inhibitor, after completion of 2 cycles and tolerating well with no significant adverse events	
Adjuvant zoledronic acid +/− ET	
Adjuvant ET without primary-care provider and patient cannot be discharged	

Abbreviations: CDK, cyclin dependent kinase; ECOG PS = Eastern Cooperative Oncology Group Performance Status; ET = endocrine therapy; T-DM1 = ado-trastuzumab emtansine.

**Table 2:** Recommendations for implementing GPO/NP-led innovative care models.

Category	Recommendations
Referral Pathways and Risk Criteria	- Establish clear, standardized referral pathways between oncologists and innovative care model providers (e.g., GPO, NP). - Define patient eligibility and risk stratification criteria. - Use EMR systems for seamless communication and documentation. - Employ structured transfer-of-care letters.
Patient Education	- Provide comprehensive educational materials. - Direct patients to resources like cancer navigation services and organizations (e.g., Wellspring). - Encourage self-care and prompt symptom reporting.
Clinic Structure and Follow-Up	- Standardize clinic structure and follow-up schedules. - Focus appointments on adherence, toxicity management, and recurrence surveillance. - Use virtual visits as needed. - Monitor for complications and make specialist referrals as necessary.
Workflow and Resource Optimization	- Optimize workflow and resource allocation. - Ensure institutional funding for clinic space, resources, and staffing. - Maintain continuity of care with consistent nursing staff. - Implement scalable patient volume management.
Quality Assessment	- Implement quality assessment measures. - Collect PROs for satisfaction, adherence, QoL, and symptom management. - Monitor provider satisfaction. - Track health resource utilization for cost-effectiveness.

Abbreviations: GPO = general practitioner in oncology; NP = nurse practitioner; PROs, patient-reported outcomes; QoL = quality of life.

**Table 3:** Recommendations for implementing pharmacist-led care models.

Category	Recommendations
Scope and Training	- Clearly outline pharmacists’ roles within oncology teams. - Provide comprehensive training in oncology pharmacology, toxicity management, and patient communication.
Proactive Care Interventions	- Implement proactive telephone assessments. - Ensure consistent patient education to improve care quality and reduce reactive visits.
Collaboration	- Enhance teamwork with oncologists, nurses, and other healthcare professionals. - Secure clinic space near the multidisciplinary clinic setting. - Ensure seamless care transitions and comprehensive patient support. - Alternate follow-ups between pharmacists and physicians. - Coordinate with a physician to order tests if not within scope of practice.
Infrastructure and Support	- Invest in clinic space, digital tools, and streamlined workflows. - Ensure adequate clerical support. - Integrate telehealth, EMR, and patient portals for better accessibility.
Quality Assessment	- Use standardized metrics like adherence rates and patient satisfaction. - Identify quality improvements and demonstrate clinical value.
Expanded Scope	- Broaden pharmacist-led models to community settings with secured funding. - Expand prescribing authority for supportive care medications and OAMs.

Abbreviations: EMR = electronic medical record; OAMs = oral anticancer medications.

**Table 4:** Recommendations for implementing digital symptom-monitoring tools.

Recommendation	Description
Cultural Shift	- Promote a healthcare culture that emphasizes proactive symptom management alongside patient empowerment.
Dedicated Resources	- Secure organizational backing to provide the necessary support and resources for long-term success. - Allocate financial and human resources to create a role for a clinical champion in symptom monitoring.
Technology Implementation	- Implement symptom reporting questionnaires that capture adverse-event grades to ensure appropriate and timely clinical interventions. - Allow individual centers to tailor the monitoring model to their unique clinical environments. - Ensure patients have access to user-friendly technology for seamless system engagement.
Continuous Evaluation	- Conduct ongoing research to refine and optimize the monitoring system for evolving needs.

**Table 5:** Summary of innovative care models.

Care Model	Funding	Referral	Services ^a^	Strengths	Limitations
Nurse/NP-led	- Provider: Institution or health authority - Resources: ^b^ Institution (e.g., for training, clinic space, administrative staff)	- Oncologist prescribes and initiates therapy - Some models transition patients early (first cycle), others when stable (e.g., no grade ≥ 2 AEs)	- Education - F/u care - Toxicity management - NPs: Dose adjustments - Prescriptions (refills) Survivorship care	- Wide scope of practice (e.g., medication dose adjustments, prescribe refills) - Longitudinal support for adherence, especially with endocrine agents	- Shortage of trained NPs - Limitations on prescribing for nurses - Limitations on scope of practice for nurses
GPO-led	- Provider: Institution, health authority or provincial budget - Resources: ^b^ Institution	- Oncologist prescribes and initiates therapy - Some models transition patients early (first cycle), others when patients are stable (e.g., no grade ≥ 2 AEs)	- Education - F/u care - Toxicity management - Dose adjustments - Prescriptions (refills) - Survivorship care	- Wide scope of practice (e.g., medication dose adjustments, prescribe refills) - Effective bridge to primary care - May be cost-effective if funded by province	- Shortage of trained GPOs
Pharmacist-led	- Provider: Institution or health authority; in some centers, revenue from dispensing medications - Resources: ^b^ Institution or revenue from pharmacy	- Oncologist prescribes and initiates therapy - Patient consults with pharmacist at treatment onset - Some models transition patients early (first cycle), others when stable (e.g., no grade ≥ 2 AEs)	E ducation - F/u care - Medication reviews - Toxicity management - Adherence checks - Reimbursement navigation	- Experience with OAMs - Strong DDI detection capacity - Expertise in polypharmacy contexts	- Staff shortages - Limitations on prescribing - Limitations on conducting physical exams and ordering diagnostic imaging - Variable referral processes
Digital health tools	- Institution (e.g., for digital platforms, tech support, staff to monitor alerts) and/or grants (e.g., for pilot projects)	- Patient opt-in	- Symptom monitoring - Self-management tools	- Early detection of symptoms - Early clinical interventions - Reduced ER visits - Aggregate data	- Patient opt-in - Limited access for some patients (e.g., elderly, restricted digital literacy, socioeconomic reasons)

^a^ The specific services provided will vary depending on each institution. ^b^ Resources include the funding for training staff, for clinic space, and for hiring administrative staff to support the clinic. Abbreviations: AEs = adverse events; DDI = drug-drug interaction; ER = emergency room; GF/u = follow-up; PO = general practitioner in oncology; OAMs = oral anticancer medications; NP = nurse practitioner.

## Data Availability

Data are contained within the article.

## Abbreviations

The following abbreviations are used in this manuscript:

AHS	Alberta Health Services
ASCO	American Society of Clinical Oncology
BCTH	Bloods closer to home
cpKPI	Clinical pharmacy key performance indicator
DRP	Drug-related problem
EBC	Early breast cancer
EMR	Electronic medical record
ER	Emergency room
GPO	General practitioner in oncology
HER2–	Human epidermal growth factor receptor 2-negative
HR+	Hormone receptor-positive
HRQoL	Health-related quality of life
ITT	Intent-to-treat
MAP	Medication Assessment by Pharmacists
NCODA	National Community Oncology Dispensing Association
NP	Nurse practitioner
OAM	Oral anticancer medication
OFS	Ovarian function suppression
PDSA	Plan-do-study-act
PEPPA	Participatory, evidence-based, patient-focused process for advanced practice nursing
PRO	Patient-reported outcome
SARO-MAVO	Suivi Actif des Résultats rapportés par le patient en Oncologie—Médicaments Anti-néoplasiques administrés par Voie Orale
SCP	Survivorship care plan
SURC	Symptom and urgent review clinic
